# Supplementary material for: Extending the MR‐Egger method for multivariable Mendelian randomization to correct for both measured and unmeasured pleiotropy
Source: Stat Med. 2017 Sep 27;36(29):4705–18. doi: 10.1002/sim.7492 (PMC5725762; doi:10.1002/sim.7492)
Supplement: Supplementary file 1 — SIM_7492‐Sup‐0001‐Rees_appendix.pdf [file SIM-36-4705-s001.pdf]

Web Appendix

A1 Sample software code

We provide sample code written in R to perform the analyses described in this paper. The associations of the genetic variants with the risk factors are denoted `bX`*k* with standard error `bX`*k*`se`, where *k* `= 1, ..., K`. The associations of the genetic variants with the outcome are denoted `bY` with standard error `bYse`. The code for the multivariable models will be based on three risk factors and can be easily adapted to include the appropriate number of risk factors. It will be assumed that the causal effect of risk factor 1 on the outcome is of primary interest and all the genetic variants are uncorrelated.

Inverse-variance weighted estimate:

The inverse-variance weighted (IVW) estimate using summary statistics (equation 2) can be calculated by:

```
thetaUI     = sum(bY*bX1*bYse^-2)/sum(bX1^2*bYse^-2)
se_thetaUI  = 1/sqrt(sum(bX1^2*bYse^-2))
```

The same IVW estimate using summary statistics can be obtained using weighted linear regression (equation 3):

```
thetaUI            = summary(lm(bY~bX1-1, weights=bYse^-2))$coef[1]
se_thetaUI.fixed   = summary(lm(bY~bX1-1, weights=bYse^-2))$coef[1,2]/
                       summary(lm(bY~bX1-1, weights=bYse^-2))$sigma
se_thetaUI.random  = summary(lm(bY~bX1-1, weights=bYse^-2))$coef[1,2]/
                       min(summary(lm(bY~bX1-1, weights=bYse^-2))$sigma,1)
```

In the fixed-effect model we divide the standard error of the causal estimate by the estimated residual standard error to force the residual standard error to be 1. For the multiplicative random-effect model the standard error is divided by the estimated residual standard error when the variability in the genetic associations is less than expected by chance (underdispersion). When there is evidence of heterogeneity between the causal estimates (overdispersion) the standard error is unaltered. The multiplicative random-effects model will result in a larger standard error compared to the fixed-effect model if there is heterogeneity between the causal estimates. The causal estimate obtained from the fixed- and multiplicative random-effects models will be the same.

Univariable MR-Egger:

The univariable MR-Egger method is the same as the IVW method using weighted linear regression except the intercept term is estimated rather than being set to zero. Testing whether the intercept term is equal to zero is equivalent to testing for directional pleiotropy and the validity of the InSIDE assumption. The genetic associations with the risk factor `bX1` and outcome `bY` must be orientated with respect to the risk increasing or decreasing allele of the risk factor. Under the MR-Egger model, multiplicative random-effects should be used as the presence of pleiotropy will lead to overdispersion. Since the residual standard error is estimated, we use the t-distribution with $J - 2$ degrees of freedom for inference.

```
#Orientation of the genetic associations
bY<-ifelse(bX1>0, bY, bY*-1)
bX1<-abs(bX1)
#Causal estimate
thetaUE            = summary(lm(bY~bX1, weights=bYse^-2))$coef[2]
se_thetaUE.random  = summary(lm(bY~bX1, weights=bYse^-2))$coef[2,2]/
                         min(summary(lm(bY~bX1, weights=bYse^-2))$sigma,1)
lb_thetaUE         = thetaUE - qt(0.975,df=length(bX1)-2)*se_thetaUE.random
ub_thetaUE         = thetaUE + qt(0.975,df=length(bX1)-2)*se_thetaUE.random
p_thetaUE          = 2*(1-pt(abs(thetaUE/se_thetaUE.random),df=length(bX1)-2))
#Test for directional pleiotropy
interUE            = summary(lm(bY~bX1, weights=bYse^-2))$coef[1]
se_interUE.random  = summary(lm(bY~bX1, weights=bYse^-2))$coef[1,2]/
                         min(summary(lm(bY~bX1, weights=bYse^-2))$sigma,1)
p_interUE          = 2*(1-pt(abs(interUE/se_interUE.random),df=length(bX1)-2))
```

Multivariable IVW:

The multivariable IVW method expands the IVW method using weighted linear regression by estimating the causal effects of the additional risk factors on the outcome. We will include additional two risk factors and assume the causal estimate of interest is the effect of risk factor 1 on the outcome. Either fixed- or multiplicative random-effects can be used to estimate the standard error of the causal effect.

```
theta1MI            = summary(lm(bY~bX1+bX2+bX3-1, weights=bYse^-2))$coef[1]
se_theta1MI.fixed   = summary(lm(bY~bX1+bX2+bX3-1, weights=bYse^-2))$coef[1,2]/
                          summary(lm(bY~bX1+bX2+bX3-1, weights=bYse^-2))$sigma
se_theta1MI.random  = summary(lm(bY~bX1+bX2+bX3-1, weights=bYse^-2))$coef[1,2]/
                          min(summary(lm(bY~bX1+bX2+bX3-1, weights=bYse^-2))$sigma,1)
```

Multivariable MR-Egger:

The multivariable MR-Egger method is equivalent to the multivariable IVW method using weighted linear regression except the intercept is estimated rather than being set to zero. Testing whether the intercept term is equal to zero is equivalent to testing

for directional pleiotropy and the validity of the InSIDE assumption. As with univariable MR-Egger, the standard errors should be calculated from the multiplicative random-effects model. The genetic associations should be orientated with respect to the risk increasing or decreasing allele of the risk factor of interest. In this sample code we will assume the causal effect of risk factor 1 is of primary interest. Since the residual standard error is estimated for the multivariable MR-Egger model we use the t-distribution with $J-(K+1)$ degrees of freedom for inference.

```
#Orientation of the genetic associations with respect to X1
clist<-c("bX2","bX3","bY")
for (var in clist){
  eval(parse(text=paste0(var,"<-ifelse(bX1>0,",var,",",var,"*-1)")))
}
bX1<-abs(bX1)
#Causal estimate for X1
theta1ME            = summary(lm(bY~bX1+bX2+bX3, weights=bYse^-2))$coef[2]
se_theta1ME.random  = summary(lm(bY~bX1+bX2+bX3, weights=bYse^-2))$coef[2,2]/
                          min(summary(lm(bY~bX1+bX2+bX3, weights=bYse^-2))$sigma,1)
lb_theta1ME         = theta1ME - qt(0.975,df=length(bX1)-4)*se_theta1ME.random
ub_theta1ME         = theta1ME + qt(0.975,df=length(bX1)-4)*se_theta1ME.random
p_theta1ME          = 2*(1-pt(abs(theta1ME/se_theta1ME.random),df=length(bX1)-4))
#Test for directional pleiotropy
interME            = summary(lm(bY~bX1+bX2+bX3, weights=bYse^-2))$coef[1]
se_interME.random  = summary(lm(bY~bX1+bX2+bX3, weights=bYse^-2))$coef[1,2]/
                          min(summary(lm(bY~bX1+bX2+bX3, weights=bYse^-2))$sigma,1)
p_interME          = 2*(1-pt(abs(interME/se_interME.random),df=length(bX1)-4))
```

A2 Comparison between the precision of the causal estimates from univariable and multivariable MR-Egger

In this section, we compare the precision of the causal estimates from the univariable ($\hat{\theta}_{1UE}$) and multivariable ($\hat{\theta}_{1ME}$) MR-Egger models. For the multivariable model, we consider the genetic associations $\boldsymbol{\beta_{X_k}}$ with two risk factors ($k = 2$), where the variance of the multivariable MR-Egger estimate $\hat{\theta}_{1ME}$ is given by:

$$\begin{aligned}\operatorname{var}(\hat{\theta}_{1ME}) &= \frac{\phi^2 \operatorname{var}(\boldsymbol{\beta_{X_2}})}{N(\operatorname{var}(\boldsymbol{\beta_{X_1}}) \operatorname{var}(\boldsymbol{\beta_{X_2}}) - \operatorname{cov}(\boldsymbol{\beta_{X_1}}, \boldsymbol{\beta_{X_2}})^2)} \\ &\propto [\operatorname{var}(\boldsymbol{\beta_{X_1}})(1 - \operatorname{cor}(\boldsymbol{\beta_{X_1}}, \boldsymbol{\beta_{X_2}})^2)]^{-1} \end{aligned} \quad (1)$$

Under a fixed-effect model, the variance of the univariable MR-Egger estimate is proportional to the inverse of $\operatorname{var}(\boldsymbol{\beta_{X_1}})$.[1] The estimate from the multivariable MR-Egger model $\hat{\theta}_{1ME}$ will be more precise than its univariable counterpart $\hat{\theta}_{1UE}$ if:

$$\frac{1}{\operatorname{var}(\boldsymbol{\beta_{X_1}})} > \frac{1}{\operatorname{var}(\boldsymbol{\beta_{X_1}})(1 - \operatorname{cor}(\boldsymbol{\beta_{X_1}}, \boldsymbol{\beta_{X_2}})^2)} \quad (2)$$

From the above inequality, $\hat{\theta}_{1UE}$ will always be more precise than $\hat{\theta}_{1ME}$ when $\boldsymbol{\beta_{X_1}}$ and $\boldsymbol{\beta_{X_2}}$ are correlated. Under a multiplicative random-effects model (used throughout this paper), the variance of the residual error is estimated under the univariable MR-Egger model (ϕ^2_{UE}) and the multivariable MR-Egger model (ϕ^2_{ME}). For $\hat{\theta}_{1ME}$ to be more precise than $\hat{\theta}_{1UE}$, we require:

$$\frac{\phi^2_{UE}}{\operatorname{var}(\boldsymbol{\beta_{X_1}})} > \frac{\phi^2_{ME}}{\operatorname{var}(\boldsymbol{\beta_{X_1}})(1 - \operatorname{cor}(\boldsymbol{\beta_{X_1}}, \boldsymbol{\beta_{X_2}})^2)} \quad (3)$$

If $\boldsymbol{\beta_{X_2}}$ explains additional independent variability in the genetic associations with the outcome $\boldsymbol{\beta_Y}$, and $\boldsymbol{\beta_{X_1}}$ and $\boldsymbol{\beta_{X_2}}$ are independent, then the estimate from multivariable MR-Egger will be more precise than the estimate from univariable MR-Egger. If $\boldsymbol{\beta_{X_1}}$ and $\boldsymbol{\beta_{X_2}}$ are correlated, then the precision of $\hat{\theta}_{1ME}$ will depend upon the strength of the correlation between $\boldsymbol{\beta_{X_1}}$ and $\boldsymbol{\beta_{X_2}}$, and the amount of additional independent variability $\boldsymbol{\beta_{X_2}}$ explains in $\boldsymbol{\beta_Y}$. As the correlation between $\boldsymbol{\beta_{X_1}}$ and $\boldsymbol{\beta_{X_2}}$ increases, and $\boldsymbol{\beta_{X_2}}$ explains no additional independent variability in $\boldsymbol{\beta_Y}$, the precision of the multivariable MR-Egger estimate $\hat{\theta}_{1ME}$ will decrease.

A3 Summary statistics from the simulation study

The IVW and MR-Egger methods do not account for uncertainty in the genetic associations with the risk factor, referred to by Bowden et al as NO Measurement Error (NOME).[1] If there is substantial uncertainty in these association estimates and in a two-sample setting, the causal effect estimate from univariable MR-Egger may be biased towards the null. Bowden et al have shown that the relative attenuation in the MR-Egger estimate is approximately equal to the I^2 statistic from the meta-analysis of the weighted associations with the exposure $\hat{\beta}_{Xj}\,\mathrm{se}(\hat{\beta}_{Yj})^{-1}$, with standard errors $\mathrm{se}(\hat{\beta}_{Xj})\,\mathrm{se}(\hat{\beta}_{Yj})^{-1}$.[1] The I^2 statistic lies between 0 and 1, with smaller values corresponding to more biased MR-Egger estimates. If the I^2 statistic is close to 1, then there should be little or no attenuation of the causal estimate from the univariable MR-Egger method. Bowden et al recommend that methods to account for this uncertainty be considered if the I^2 statistic is less than 90%.[1]

The F-statistic is often reported in Mendelian randomization studies as a measurement of the strength of the instrumental variables, with larger values representing stronger instruments. For a two-sample Mendelian randomization analysis with summarized data, the F-statistic for each genetic variant j can be approximated by $F_j = \hat{\beta}^2_{X_j} / \,\mathrm{se}(\hat{\beta}_{X_j})^2$. We use this approximation below.

The data-generating model used in the simulation study did not provide the standard errors of the genetic associations with the three risk factors $\mathrm{se}(\hat{\boldsymbol{\beta}}_{\boldsymbol{X_k}})$, as they were not required for the methods considered. To estimate the mean values of the F-statistics and I^2 statistics, we must make assumptions about the values of these standard errors. We assume that the genetic associations with the risk factors are provided on the standard deviation scale. If the associations were estimated from a sample size of 10 000, this results in a standard error of 0.01. Assuming that the standard errors of the genetic associations with the three risk factors are 0.01 across the 185 genetic variants, we obtain the mean F-statistics and I^2 statistics displayed in Table A1 and Table A2. The I^2 statistics (reported as a %) are close to 100% across the different scenarios. These results are consistent with the simulation study where the causal estimates from the univariable and multivariable MR-Egger methods showed no attenuation towards the null.

Table A1: Mean F-statistic and I^2 statistic (reported as a %) for a null ($\theta_1 = 0$) and positive ($\theta_1 = 0.3$) causal effect where $\boldsymbol{\beta_{X_k}}$ are generated independently for all k.

	$\hat{\beta}_{X_{1j}}$		$\hat{\beta}_{X_{2j}}$		$\hat{\beta}_{X_{3j}}$	
	F-statistic	I^2 statistic	F-statistic	I^2 statistic	F-statistic	I^2 statistic
**Null causal effect: $\boldsymbol{\theta_1 = 0}$**						
1. No pleiotropy, InSIDE satisfied						
	363.3	99.5	208.8	99.2	425.7	99.6
2. Balanced pleiotropy, InSIDE satisfied						
$\alpha'_j \sim \mathcal{N}(0,0.004)$	364.3	99.5	209.1	99.2	425.6	99.6
3. Directional pleiotropy, InSIDE satisfied						
$\alpha'_j \sim \mathcal{N}(0.01,0.004)$	364.4	99.5	208.9	99.2	425.6	99.6
$\alpha'_j \sim \mathcal{N}(0.05,0.004)$	363.5	99.5	209.6	99.2	424.9	99.6
$\alpha'_j \sim \mathcal{N}(0.1,0.004)$	364.0	99.5	209.2	99.2	425.5	99.6
4. Directional pleiotropy, InSIDE violated						
$\alpha'_j \sim \mathcal{N}(0.01,0.004)$	364.1	99.5	208.8	99.2	425.4	99.6
$\alpha'_j \sim \mathcal{N}(0.05,0.004)$	364.4	99.5	208.7	99.2	425.4	99.6
$\alpha'_j \sim \mathcal{N}(0.1,0.004)$	363.8	99.5	209.1	99.2	425.1	99.6
**Positive causal effect: $\boldsymbol{\theta_1 = 0.3}$**						
1. No pleiotropy, InSIDE satisfied						
	363.9	99.5	209.2	99.2	424.7	99.6
2. Balanced pleiotropy, InSIDE satisfied						
$\alpha'_j \sim \mathcal{N}(0,0.004)$	363.7	99.5	209.1	99.2	425.0	99.6
3. Directional pleiotropy, InSIDE satisfied						
$\alpha'_j \sim \mathcal{N}(0.01,0.004)$	364.1	99.5	209.0	99.2	425.2	99.6
$\alpha'_j \sim \mathcal{N}(0.05,0.004)$	364.3	99.5	208.6	99.2	425.5	99.6
$\alpha'_j \sim \mathcal{N}(0.1,0.004)$	363.8	99.5	209.1	99.2	424.7	99.6
4. Directional pleiotropy, InSIDE violated						
$\alpha'_j \sim \mathcal{N}(0.01,0.004)$	363.6	99.5	209.1	99.2	424.8	99.6
$\alpha'_j \sim \mathcal{N}(0.05,0.004)$	364.6	99.5	208.9	99.2	424.8	99.6
$\alpha'_j \sim \mathcal{N}(0.1,0.004)$	364.0	99.5	208.9	99.2	425.9	99.6

Table A2: Mean F-statistic and I^2 statistic (reported as a %) for a null ($\theta_1 = 0$) and positive ($\theta_1 = 0.3$) causal effect with $\boldsymbol{\beta_{X_k}}$ being correlated for all k.

	$\hat{\beta}_{X_{1j}}$		$\hat{\beta}_{X_{2j}}$		$\hat{\beta}_{X_{3j}}$	
	F-statistic	I^2 statistic	F-statistic	I^2 statistic	F-statistic	I^2 statistic
**Null causal effect: $\boldsymbol{\theta_1 = 0}$**						
1. No pleiotropy, InSIDE satisfied						
	364.1	99.5	208.5	99.2	424.5	99.6
2. Balanced pleiotropy, InSIDE satisfied						
$\alpha'_j \sim \mathcal{N}(0,0.004)$	363.8	99.5	209.4	99.2	424.4	99.6
3. Directional pleiotropy, InSIDE satisfied						
$\alpha'_j \sim \mathcal{N}(0.01,0.004)$	363.5	99.5	208.8	99.2	424.6	99.6
$\alpha'_j \sim \mathcal{N}(0.05,0.004)$	364.3	99.5	209.0	99.2	425.1	99.6
$\alpha'_j \sim \mathcal{N}(0.1,0.004)$	364.3	99.5	208.9	99.2	424.7	99.6
4. Directional pleiotropy, InSIDE violated						
$\alpha'_j \sim \mathcal{N}(0.01,0.004)$	364.0	99.5	208.9	99.2	425.0	99.6
$\alpha'_j \sim \mathcal{N}(0.05,0.004)$	364.0	99.5	209.4	99.2	425.2	99.6
$\alpha'_j \sim \mathcal{N}(0.1,0.004)$	364.3	99.5	209.1	99.2	425.2	99.6
**Positive causal effect: $\boldsymbol{\theta_1 = 0.3}$**						
1. No pleiotropy, InSIDE satisfied						
	364.0	99.5	208.9	99.2	425.2	99.6
2. Balanced pleiotropy, InSIDE satisfied						
$\alpha'_j \sim \mathcal{N}(0,0.004)$	364.0	99.5	208.8	99.2	425.1	99.6
3. Directional pleiotropy, InSIDE satisfied						
$\alpha'_j \sim \mathcal{N}(0.01,0.004)$	363.5	99.5	209.1	99.2	425.5	99.6
$\alpha'_j \sim \mathcal{N}(0.05,0.004)$	363.9	99.5	209.1	99.2	424.6	99.6
$\alpha'_j \sim \mathcal{N}(0.1,0.004)$	364.0	99.5	209.1	99.2	425.8	99.6
4. Directional pleiotropy, InSIDE violated						
$\alpha'_j \sim \mathcal{N}(0.01,0.004)$	364.1	99.5	208.8	99.2	425.3	99.6
$\alpha'_j \sim \mathcal{N}(0.05,0.004)$	364.7	99.5	208.8	99.2	425.4	99.6
$\alpha'_j \sim \mathcal{N}(0.1,0.004)$	363.7	99.5	208.9	99.2	424.5	99.6

A4 Details and results from the simulation study investigating causal relationships between risk factors

To investigate the behaviour of the multivariable MR-Egger method when causal relationships between risk factors exist, additional simulations were performed where X_2 was causally dependent on X_1. We assume that X_2 is causally dependent on X_1, and the causal effect of X_1 on X_2 is γ. The total causal effect of X_1 on Y is $\theta_1 + \gamma\theta_2$; consisting of the direct effect (θ_1) and the indirect effect via X_2 ($\gamma\theta_2$). The simulations outlined in Section 4 were repeated with the second line in the data generating model replaced with:

$$\beta_{Y_j} = \alpha'_j + \theta_1|\beta_{X_{1j}}| + \theta_2(\beta_{X_{2j}} + \gamma|\beta_{X_{1j}}|) + \theta_3\beta_{X_{3j}} + \epsilon_j \quad (4)$$

The causal effect of X_1 on X_2 (γ) was set to 0.5. All other parameters were taken as in the original simulation study. $|\beta_{X_{1j}}|$, $(\beta_{X_{2j}} + \gamma|\beta_{X_{1j}}|)$, and $\beta_{X_{3j}}$ were the covariates included in the multivariable IVW and multivariable MR-Egger models. Note that the functional relationship between X_1 and X_2 induces a correlation structure between the covariates $|\beta_{X_{1j}}|$ and $(\beta_{X_{2j}} + \gamma|\beta_{X_{1j}}|)$ included in the multivariable models, even when $\boldsymbol{\beta_{X_1}}$ and $\boldsymbol{\beta_{X_2}}$ are generated independently. To account for the additional uncertainty in β_{Y_j}, the weights for univariable MR-Egger are given by equation 5, while the weights for multivariable IVW and multivariable MR-Egger were the same as the original simulation study (equation 15).

$$\text{se}(\beta_{Y_j})^{-2} = ({\epsilon_j}^2 + {\sigma_{\alpha'}}^2 + {\theta_2}^2{\sigma_2}^2 + (\theta_2\gamma)^2{\sigma_1}^2 + 2\theta_2\gamma\rho_{12}\sigma_1\sigma_2 + {\theta_3}^2{\sigma_3}^2)^{-1} \quad (5)$$

Results

The results from the simulations that included a causal relationship between X_1 and X_2, using 10 000 simulated datasets, are presented in Web Table A3 ($\boldsymbol{\beta_{X_k}}$ generated independently, with the functional relationship between X_1 and X_2 inducing a correlation structure between $|\beta_{X_{1j}}|$ and $(\beta_{X_{2j}}+\gamma|\beta_{X_{1j}}|)$) and Web Table A4 ($\boldsymbol{\beta_{X_k}}$ correlated).

$\boldsymbol{\beta_{X_k}}$ generated independently, with a correlation structure between the covariates $|\beta_{X_{1j}}|$ and $(\beta_{X_{2j}} + \gamma|\beta_{X_{1j}}|)$: In scenarios where there was no bias in the original set of simulations, the multivariable IVW and multivariable MR-Egger methods consistently estimated the direct effect of X_1 on Y (θ_1), whilst the univariable MR-Egger method consistently estimated the total causal effect of X_1 on Y ($\theta_1 + \gamma\theta_2$).

Bias for the multivariable IVW method was present in scenarios 3 and 4 only, as in the original simulation study (Tables 3 and 4). Compared to the results in Table 3, precision and power to detect a causal effect were reduced for the multivariable IVW and multivariable MR-Egger methods. This reduction in power may be due to the correlation structure between $|\beta_{X_{1j}}|$ and $(\beta_{X_{2j}} + \gamma|\beta_{X_{1j}}|)$, and the multivariable models conditioning on a mediator. Univariable and multivariable MR-Egger methods produced biased estimates of the total and direct causal effects in scenario 4 (InSIDE violated) only. Unlike the original simulation study, precision and power to detect a causal effect were always better for the univariable MR-Egger method.

$\boldsymbol{\beta_{X_k}}$ correlated: The multivariable IVW and multivariable MR-Egger methods estimated the direct effect of X_1 on Y, as in the independently generated setting. As with the original simulations (Tables 3 and 4), the InSIDE assumption for univariable MR-Egger was violated for all four scenarios, resulting in biased point estimates. However, as with the original simulation study, the multivariable InSIDE assumption was satisfied for scenarios 1,2 and 3, and so causal estimates from multivariable MR-Egger were unbiased. There was a more noticeable reduction in the precision and power to detect a causal effect for the multivariable IVW and multivariable MR-Egger methods under the correlated setting.

Table A3: Performance of multivariable IVW, univariable MR-Egger and multivariable MR-Egger with respect to $\hat{\theta}_1$ for a null ($\theta_1 = 0$) and positive ($\theta_1 = 0.3$) causal effect where $\boldsymbol{\beta_{X_k}}$ are generated independently for all k (with a correlation structure between the covariates $|\beta_{X_{1j}}|$ and $(\beta_{X_{2j}} + \gamma|\beta_{X_{1j}}|)$), with a causal effect of X_1 on X_2 ($\gamma = 0.5$). All tests were performed at the 5% level of significance.

	**Multivariable IVW**		**Univariable MR-Egger**			**Multivariable MR-Egger**		
	Mean $\hat{\theta}_1$ (mean SE)	Power, %	Mean $\hat{\theta}_1$ (mean SE)	Power, % Intercept	Causal	Mean $\hat{\theta}_1$ (mean SE)	Power, % Intercept	Causal
**Null causal effect: $\boldsymbol{\theta_1 = 0}$**								
1. No pleiotropy, InSIDE satisfied								
	0.000 (0.057)	3.5	0.051 (0.158)	8.9	5.8	0.001 (0.090)	4.5	4.2
2. Balanced pleiotropy, InSIDE satisfied								
$\alpha'_j \sim \mathcal{N}(0,0.004)$	0.001 (0.127)	4.4	0.049 (0.187)	7.6	5.6	0.001 (0.178)	4.6	4.2
3. Directional pleiotropy, InSIDE satisfied								
$\alpha'_j \sim \mathcal{N}(0.01,0.004)$	0.041 (0.127)	6.0	0.049 (0.187)	12.3	5.4	0.000 (0.178)	5.8	4.8
$\alpha'_j \sim \mathcal{N}(0.05,0.004)$	0.195 (0.128)	34.4	0.048 (0.187)	50.1	5.3	-0.001 (0.178)	36.6	4.6
$\alpha'_j \sim \mathcal{N}(0.1,0.004)$	0.393 (0.130)	82.3	0.052 (0.187)	91.4	5.6	0.002 (0.178)	88.4	4.7
4. Directional pleiotropy, InSIDE violated								
$\alpha'_j \sim \mathcal{N}(0.01,0.004)$	0.076 (0.127)	9.8	0.138 (0.187)	6.4	11.6	0.088 (0.178)	4.3	7.6
$\alpha'_j \sim \mathcal{N}(0.05,0.004)$	0.231 (0.127)	45.2	0.137 (0.187)	34.4	11.9	0.088 (0.178)	21.7	8.2
$\alpha'_j \sim \mathcal{N}(0.1,0.004)$	0.426 (0.129)	88.3	0.141 (0.187)	83.7	11.9	0.089 (0.178)	78.2	8.1
**Positive causal effect: $\boldsymbol{\theta_1 = 0.3}$**								
1. No pleiotropy, InSIDE satisfied								
	0.301 (0.057)	96.3	0.353 (0.158)	9.3	62.3	0.301 (0.090)	3.9	84.6
2. Balanced pleiotropy, InSIDE satisfied								
$\alpha'_j \sim \mathcal{N}(0,0.004)$	0.298 (0.127)	65.4	0.350 (0.187)	7.4	47.8	0.298 (0.178)	4.4	41.2
3. Directional pleiotropy, InSIDE satisfied								
$\alpha'_j \sim \mathcal{N}(0.01,0.004)$	0.338 (0.127)	75.5	0.352 (0.187)	11.8	48.3	0.300 (0.178)	6.1	41.1
$\alpha'_j \sim \mathcal{N}(0.05,0.004)$	0.494 (0.128)	95.2	0.348 (0.188)	49.2	46.9	0.298 (0.179)	36.8	40.3
$\alpha'_j \sim \mathcal{N}(0.1,0.004)$	0.689 (0.130)	99.6	0.347 (0.188)	91.5	47.1	0.296 (0.178)	88.2	39.6
4. Directional pleiotropy, InSIDE violated								
$\alpha'_j \sim \mathcal{N}(0.01,0.004)$	0.375 (0.127)	82.6	0.440 (0.187)	6.6	65.7	0.390 (0.178)	4.7	60.1
$\alpha'_j \sim \mathcal{N}(0.05,0.004)$	0.530 (0.128)	97.0	0.438 (0.187)	34.7	65.5	0.386 (0.178)	21.7	59.9
$\alpha'_j \sim \mathcal{N}(0.1,0.004)$	0.728 (0.129)	99.7	0.441 (0.187)	83.6	65.8	0.390 (0.178)	78.5	60.1

Abbreviations: MR, Mendelian randomization; SE, standard error; IVW, inverse-variance weighted; InSIDE, Instrument Strength Independent of Direct Effect.

Table A4: Performance of multivariable IVW, univariable MR-Egger and multivariable MR-Egger with $\boldsymbol{\beta}_{\boldsymbol{X_k}}$ being correlated for all k, and a causal effect of X_1 on X_2

	**Multivariable IVW**		**Univariable MR-Egger**			**Multivariable MR-Egger**		
	Mean $\hat{\theta}_1$ (mean SE)	Power, %	Mean $\hat{\theta}_1$ (mean SE)	Power, % Intercept	Causal	Mean $\hat{\theta}_1$ (mean SE)	Power, % Intercept	Causal
**Null causal effect: $\boldsymbol{\theta_1 = 0}$**								
1. No pleiotropy, InSIDE satisfied								
	0.000 (0.062)	4.1	0.146 (0.158)	3.9	15.6	0.000 (0.097)	4.0	4.0
2. Balanced pleiotropy, InSIDE satisfied								
$\alpha'_j \sim \mathcal{N}(0,0.004)$	0.000 (0.137)	4.5	0.146 (0.188)	4.1	11.9	0.000 (0.190)	4.6	4.7
3. Directional pleiotropy, InSIDE satisfied								
$\alpha'_j \sim \mathcal{N}(0.01,0.004)$	0.041 (0.137)	5.7	0.151 (0.187)	5.4	12.8	0.003 (0.189)	5.7	4.4
$\alpha'_j \sim \mathcal{N}(0.05,0.004)$	0.209 (0.138)	34.2	0.148 (0.187)	32.8	12.6	0.000 (0.190)	36.9	4.7
$\alpha'_j \sim \mathcal{N}(0.1,0.004)$	0.422 (0.140)	82.2	0.151 (0.188)	83.0	12.9	0.004 (0.190)	89.0	4.8
4. Directional pleiotropy, InSIDE violated								
$\alpha'_j \sim \mathcal{N}(0.01,0.004)$	0.053 (0.137)	6.2	0.235 (0.188)	4.3	25.7	0.069 (0.189)	4.9	6.4
$\alpha'_j \sim \mathcal{N}(0.05,0.004)$	0.218 (0.137)	37.2	0.235 (0.188)	20.3	26.4	0.067 (0.189)	21.8	6.7
$\alpha'_j \sim \mathcal{N}(0.1,0.004)$	0.429 (0.139)	84.3	0.238 (0.188)	71.3	26.7	0.071 (0.189)	79.2	6.6
**Positive causal effect: $\boldsymbol{\theta_1 = 0.3}$**								
1. No pleiotropy, InSIDE satisfied								
	0.299 (0.062)	94.7	0.446 (0.158)	4.1	79.7	0.300 (0.096)	4.0	81.3
2. Balanced pleiotropy, InSIDE satisfied								
$\alpha'_j \sim \mathcal{N}(0,0.004)$	0.301 (0.137)	60.5	0.445 (0.187)	4.5	66.6	0.300 (0.189)	4.6	37.0
3. Directional pleiotropy, InSIDE satisfied								
$\alpha'_j \sim \mathcal{N}(0.01,0.004)$	0.339 (0.137)	69.9	0.443 (0.188)	5.7	66.1	0.296 (0.190)	6.0	36.1
$\alpha'_j \sim \mathcal{N}(0.05,0.004)$	0.510 (0.138)	94.2	0.449 (0.188)	32.6	67.7	0.302 (0.190)	37.3	37.2
$\alpha'_j \sim \mathcal{N}(0.1,0.004)$	0.715 (0.140)	99.2	0.445 (0.187)	83.4	66.9	0.298 (0.189)	89.4	36.8
4. Directional pleiotropy, InSIDE violated								
$\alpha'_j \sim \mathcal{N}(0.01,0.004)$	0.353 (0.137)	73.1	0.534 (0.188)	4.4	79.4	0.367 (0.189)	4.6	50.6
$\alpha'_j \sim \mathcal{N}(0.05,0.004)$	0.519 (0.138)	95.1	0.534 (0.188)	20.3	79.6	0.366 (0.190)	21.7	50.5
$\alpha'_j \sim \mathcal{N}(0.1,0.004)$	0.728 (0.139)	99.5	0.533 (0.188)	72.5	79.6	0.368 (0.189)	80.1	51.0

Abbreviations: MR, Mendelian randomization; SE, standard error; IVW, inverse-variance weighted; InSIDE, Instrument Strength Independent of Direct Effect.

A5 Correlated genetic variants

The methods discussed in this article have assumed that the genetic variants are uncorrelated (not in linkage disequilibrium). There may, however, be cases where using multiple correlated variants from the same gene region will be more efficient than using uncorrelated variants from different gene regions.[2] If the genetic variants are in partial linkage disequilibrium, and each variant explains independent variation in the risk factor, then the inclusion of these variants will increase the power of the MR study. The precision of a MR study will not increase, however, if the variants are perfectly correlated.

If correlated variants are included in an MR study, using summarized level data, the analysis should account for the correlation structure of the variants. If the correlation of the variants is not taken into consideration, the causal estimate will be too precise and this may lead to inappropriate inferences. To account for the correlation between the genetic variants for the univariable and multivariable IVW methods, we can use generalized weighted linear regression of the genetic associations, where the correlations of the variants are included in the weighting matrix, with the intercept set to zero.[2,3]

If $\Omega_{st} = \text{se}(\hat{\beta}_{Y_s})\,\text{se}(\hat{\beta}_{Y_t})\rho_{st}$, where ρ_{st} is the correlation between variants s and t, then the causal estimate from a weighted generalised linear regression for univariable MR is:

$$\hat{\theta}_{UIC} = (\hat{\beta}_{X_j}^T \Omega^{-1} \hat{\beta}_{X_j})^{-1} \hat{\beta}_{X_j}^T \Omega^{-1} \hat{\beta}_{Y_j} \tag{6}$$

with the standard error of the causal estimate:

$$\hat{\theta}_{UIC} = \sqrt{(\hat{\beta}_{X_j}^T \Omega^{-1} \hat{\beta}_{X_j})^{-1}} \tag{7}$$

Whilst the univariable MR-Egger estimates can be obtained by fitting the same generalized weighted linear regression model, but allowing the intercept term to be estimated, the effect of using correlated genetic variants in the univariable MR-Egger method has not been considered in detail. Further investigation into the impact correlated variants may have on the interpretation of the direct effect, and the InSIDE assumption, must be considered at the univariable level first, and then expanded to multivariable MR-Egger.

References

1. Bowden J, Del Greco M. F, Minelli C, Davey Smith G, Sheehan NA, Thompson JR. Assessing the suitability of summary data for two-sample Mendelian randomization analyses using MR-Egger regression: the role of the I2 statistic. *Int J Epidemiol.* 2016;45(6):1961–1974.

2. Burgess S, Dudbridge F, Thompson SG. Combining information on multiple instrumental variables in Mendelian randomization: comparison of allele score and summarized data methods. *Stat Med.* 2016;35(11):1880–1906.

3. Burgess S, Thompson SG. Multivariable Mendelian randomization: the use of pleiotropic genetic variants to estimate causal effects. *Am J Epidemiol.* 2015;181(4):251–260.
